# Sustained MRD Negative for 4 Years Is a Significant Marker of Prognosis in Patients with High-Risk Multiple Myeloma

**DOI:** 10.3390/cancers18101569

**Published:** 2026-05-12

**Authors:** Huan Liu, Meilan Chen, Xiaozhe Li, Lifen Kuang, Jingxia Li, Yanjuan Li, Juan Li

**Affiliations:** Department of Hematology, The First Affiliated Hospital of Sun Yat-sen University, Guangzhou 510000, China; liuh286@mail2.sysu.edu.cn (H.L.); chenmlan@mail.sysu.edu.cn (M.C.); lixzh36@mail.sysu.edu.cn (X.L.); kuanglif@mail.sysu.edu.cn (L.K.); lijx235@mail.sysu.edu.cn (J.L.); liyj355@mail.sysu.edu.cn (Y.L.)

**Keywords:** multiple myeloma, cytogenetics, minimal residual disease, duration

## Abstract

**Background/Objectives**: The optimal duration of maintaining minimal residual disease (MRD) negative status remains unclear in transplant-eligible newly diagnosed multiple myeloma (TE-NDMM). This single-center retrospective study aimed to explore the minimum duration of sustained MRD negativity with prognostic value. **Methods**: This research included 223 TE-NDMM patients who achieved at least two consecutive MRD-negative assessments after initial treatment. We analyzed the influence of sustained MRD negativity on survival in different risk stratifications. **Results**: There was no difference in progression-free survival (PFS) between high-risk (HR) patients with an MRD negativity duration of ≥2 years and patients in the standard-risk (SR) group (70.77 vs. 67.38 months, *p* = 0.529). In SR patients, PFS showed no significant difference between patients maintaining MRD negativity for 2–3 years and those maintaining MRD negativity for more than 3 years (72.25 months vs. 107.50 months, *p* = 0.103). In contrast, in the HR group, there was no difference between a duration of 4–5 years and ≥5 years (NR vs. 84.53 months, *p* = 0.136). A duration over 2 years was associated with superior overall survival (OS) for all patients. **Conclusions**: Long-term maintenance of MRD-negative status was associated with good prognosis. Sustained MRD negativity for ≥2 years was associated with reducing the adverse influence of high-risk cytogenetics, and a duration over 4 years may be correlated with longer PFS in high-risk multiple myeloma patients in an exploratory analysis based on limited cases.

## Simple Summary

Minimal residual disease (MRD) negativity is associated with superior survival in patients with multiple myeloma; however, the prognostic value of MRD negativity in high-risk multiple myeloma patients is still controversial. The purpose of this retrospective analysis is to analyze the prognostic significance of various durations of MRD negativity in high-risk multiple myeloma patients and explore the minimum duration of sustained MRD negativity that may guide individual treatment. This research enrolled 223 patients who achieved MRD negativity. We found that high-risk patients who maintained MRD negativity for 2 years had similar progression-free survival to standard-risk patients. Sustained MRD negativity for 4 years improved the poor prognosis of high-risk patients, while MRD-negative status lasting for 3 years was associated with a good prognosis in standard-risk patients.

## 1. Introduction

Multiple myeloma (MM) is one of most common hematologic malignancies in older adults and is characterized by the accumulation of monoclonal plasma cells and production of M-protein. Its typical clinical manifestations are characterized by hypercalcemia, renal insufficiency, anemia, and bone lesions, which are known as the CRAB criteria [1,2]. Recently, with the widespread use of novel drugs and application of autologous hematopoietic stem cell transplantation (ASCT), the survival of patients with multiple myeloma has been greatly prolonged. Despite most patients attaining complete remission (CR) with initial therapy, some patients still experience relapse in the short term [2,3,4]. Therefore, it is particularly urgent to optimize the response assessment criteria and risk stratification.

The prognosis of patients with multiple myeloma is determined by numerous factors, especially cytogenetic abnormalities, which have been included in risk stratification systems, such as the Revised International Staging System (R-ISS) and Mayo Stratification of Myeloma and Risk-Adapted Therapy (mSMART). Patients carrying multiple high-risk cytogenetic abnormalities (HRCA) usually experience poor outcomes [5,6,7].

Minimal residual disease (MRD) status is one of the independent and critical predictors of the survival of MM patients, and it was incorporated as a key indicator into the International Myeloma Working Group (IMWG) consensus criteria in 2016 [8,9]. Many sensitive and fast methods have been adopted for the detection of MRD, including multiparametric flow cytometry (MFC), next-generation flow (NGF) and next-generation sequencing (NGS) [8,10,11]. MRD assessment has become an important part of response evaluation and has provided guidance for developing MRD-adapted treatment strategies. Previous studies showed that MRD negativity has been established as one of the most powerful predictors of favorable clinical outcomes in MM patients; furthermore, persistent MRD-negative status is significantly linked to better progression-free survival (PFS) and overall survival (OS) [12,13,14,15,16]. Current clinical trials are attempting to suspend maintenance treatment until disease relapse in sustained MRD-negative patients or to develop MRD-driven individualized therapies [4,17,18].

Several studies have reported that attaining MRD negativity can surmount poor outcomes caused by adverse cytogenetics [19,20,21,22]. However, Kunacheewa et al. showed that high-risk cytogenetic abnormalities were one of the risk factors for survival in MRD-negative patients [23]. At present, the effect of sustained MRD negativity on the prognosis of patients with HRCAs remains controversial; moreover, there is no consensus on the duration of MRD negativity that is sufficient to guide the treatment plan and suspend maintenance treatment.

Therefore, the objective of this study is to assess the significance of durable MRD negativity for survival in patients with different cytogenetic stratifications and explore the minimum duration of sustained MRD negativity related to a good prognosis. This retrospective clinical study may provide a hypothesis for individualized treatment in MM patients. It is important to note that the ‘duration of MRD negativity’ is a complex time-dependent variable, and in retrospective analyses, this may introduce time-dependent bias.

## 2. Materials and Methods

### 2.1. Patient Selection and Treatment

This research enrolled patients with newly diagnosed multiple myeloma (NDMM) from the First Affiliated Hospital of Sun Yat-sen University between January 2003 and April 2025. All patients received induction regimens, including proteasome inhibitors (PI)-based, immunomodulatory agents (IMiD)-based, and/or daratumumab or multiple drugs combined. After induction therapy, patients underwent ASCT and subsequent maintenance treatment. The main mobilizing regimens were high-dose cyclophosphamide combined with granulocyte colony-stimulating factor and/or plerixafor. Maintenance schemes contained interferon, PIs, IMiDs, a nuclear export inhibitor (Selinexor) and/or daratumumab. During the study period, a total of 452 transplant-eligible NDMM patients were treated at our center. Among them, patients who had at least two consecutive MRD negative results and available cytogenetic data were included in this research. All enrolled patients achieved MRD negativity between 2008 and 2024. Of these, only one patient (0.4%) achieved MRD negativity in 2008, and the remaining patients (99.6%) achieved MRD negativity from 2012 to 2024. Cytogenetic abnormalities (CAs) were assessed by fluorescence in situ hybridization (FISH) at diagnosis. HRCAs were defined as deletion of 17p, t(4;14), t(14;16) and amplification of 1q (≥4 copies). Patients without any HRCA were classified as standard-risk (SR), and the high-risk (HR) group included patients with at least one HRCA. Patients were excluded if they met any of the following criteria: (1) they did not receive ASCT; (2) the result of MRD detection was positive in every test or there was a lack of continuous MRD negativity; or (3) solitary plasmacytoma progressed to MM. The collection of clinical data was based on electronic medical records. The research was approved by the Ethics Committee of the First Affiliated Hospital of Sun Yat-sen University.

### 2.2. MRD Monitoring

Since December 2010, our institution has performed serial bone marrow MRD assessment as part of routine clinical practice according to a standardized clinical protocol. MRD was evaluated by MFC at the following intervals: after induction therapy, every three months during the first year post-ASCT, and subsequently every 6 months (Figure S1). All available MRD results during the study period were used in the analysis. The assessment of bone marrow samples was performed by MFC using antibodies against the following proteins: CD19, CD20, CD38, CD45, CD54, CD56, CD138, cytoplasmic κ, and cytoplasmic λ. The number of cells detected was at least 1 × 10^6^. MRD positivity was defined as the detection of ≥20 abnormal immunophenotype plasma cells in the bone marrow aspirate, and the sensitivity of our detection method ranged from 2 × 10^−5^ to 10^−5^. MRD negativity was defined as the lack of abnormal immunophenotype plasma cells in at least two consecutive MRD assessments.

The duration of MRD negativity was considered the interval between the first MRD-negative result and the first positive result or the last negative result. In cases of missing scheduled MRD assessments, the endpoint was considered the last available negative result if the subsequent MRD assessment was negative. If the subsequent assessment was positive, the endpoint was defined as the first positive result after the missing interval. In addition, in patients who progressed between planned MRD assessments, the exact date of MRD status conversion could not be determined. The last available MRD assessment result before progression was considered the endpoint of duration.

According to IMWG definitions, sustained MRD negativity was defined as the duration of MRD negativity lasting for at least 1 year.

### 2.3. Statistical Analysis

PFS was calculated from the first negative MRD detection to progression according to IMWG response criteria, death, or the end of follow-up. OS refers to the interval from the first negative MRD detection to death or the last follow-up. Given that our primary endpoint was the minimum duration of sustained MRD negativity and the timepoints when patients achieved MRD negativity were different, we used the first MRD-negative timepoint as the start point of PFS and OS.

Statistical analyses were conducted using SPSS software (version 25.0; IBM) and R version 4.4.1. Baseline characteristics were expressed as follows: continuous variables as median (range) and categorical variables as number (percentage). For comparisons of baseline data, non-normally distributed continuous variables and categorical data were compared using the Mann–Whitney U test and chi-square test, respectively. Survival curves were analyzed by the Kaplan–Meier method and compared by the log-rank test. For pairwise comparisons involving multiple groups, the Benjamini–Hochberg (BH) procedure was used to adjust p values. In addition, to analyze factors associated with survival, univariate and multivariate Cox proportional hazards models were performed, and results were reported as hazard ratios (HR) and 95% confidence intervals (CIs). A two-sided *p* value < 0.05 was considered statistically significant.

### 2.4. Addressing Time-Dependent Bias

Because the duration of MRD negativity is inherently a time-dependent variable, bias could be introduced in retrospective analyses. We implemented the following strategies to control for time-dependent bias: (1) Landmark analysis at first MRD negativity: All survival variables (PFS and OS) were consistently calculated from the first negative MRD detection. This approach ensures a uniform starting point for all patients and reduces the bias that would arise if survival were calculated from the date of diagnosis. (2) Interval evaluation: We systematically examined the interval between the last negative and the first positive MRD assessment. The results showed that 95.9% of cases had an interval ≤12 months, and the median interval was 3.21 months. This suggested that the bias was limited. (3) To further address this bias, a time-dependent Cox regression is planned in a future prospective cohort with more precise MRD assessment data.

## 3. Results

### 3.1. Patient Characteristics

A total of 452 transplant-eligible NDMM patients were treated at our institution. Of these, 252 patients achieved MRD-negative status after initial treatment, including induction, ASCT, and maintenance therapy. Among them, 29 patients lacked continuous MRD monitoring or converted to MRD-positive status rapidly. Ultimately, 223 patients who had consecutive MRD negativity at least twice were included in the current study, and baseline characteristics are shown in Table 1. Patients had a median age of 56 years at diagnosis (range: 26–69 years) and 56.05% were male. The distribution of patients according to R-ISS stage at diagnosis was as follows: stage I, 52 (23.74%); stage II, 131 (59.82%); and stage III, 36 (16.44%). 58 patients (26.01%) carried one HRCA, and 18 patients (8.07%) had ≥2 HRCAs. All patients received ASCT, 16 of whom (7.17%) received tandem ASCT. A total of 101 patients sustained MRD-negative status for more than 2 years, including 73 patients (72.27%) in the standard-risk group, 19 patients (18.81%) with 1-HRCA, and 9 patients (8.91%) with ≥2 HRCAs. Induction regimens were mainly based on PIs (84.75%), and 62.73% of patients enrolled in our research received maintenance regimens based on IMiDs.

Patients were stratified into two subgroups by the duration of MRD negativity: duration ≥ 2 years and <2 years. The comparison of baseline data between the two groups was reported, and there was no statistically significant difference in other factors except for induction regimens (Table 2). The distribution of induction regimens differed significantly between the two groups (*p =* 0.006). Maintenance regimen distribution did not differ significantly (*p* = 0.277).

### 3.2. Survival in All Patients

**Figure 1:**
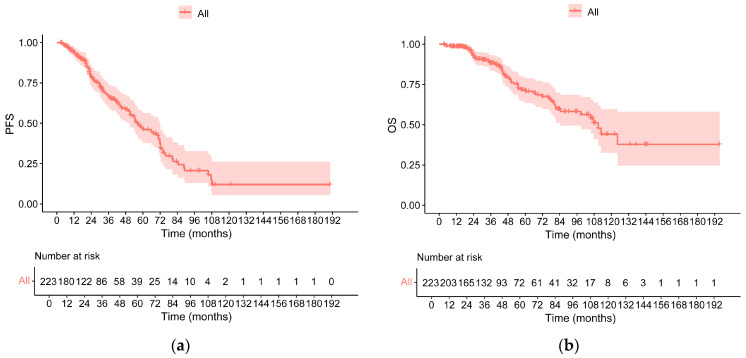
The progression-free survival (PFS) and overall survival (OS) of all enrolled patients ((**a**): PFS, (**b**): OS).

**Figure 2:**
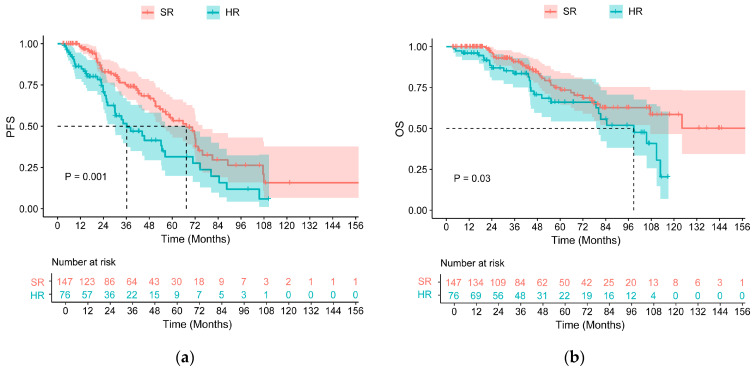
PFS (**a**) and OS (**b**) for patients in different risk groups: standard-risk (SR) and high-risk (HR).

With a median follow-up time of 54.51 (range: 3.58–195.52) months, the median PFS was 56.28 months, and the median OS was 110.82 months (Figure 1a,b). Of the 147 standard-risk patients, 53 progressed, and 31 died during follow-up, while 41 progressed, and 27 died among the 76 high-risk patients. The median PFS and OS of standard-risk patients were 67.38 months (95% CI: 52.68–82.09) and NR. The median PFS and OS were 36.17 months (95% CI: 20.33–52.02) and 98.79 months (95% CI: 71.44–126.14) in high-risk patients, respectively. Differences between the two groups were observed in both PFS (*p* = 0.001) and OS (*p* = 0.030, Figure 2a,b). In addition, the median duration of MRD negativity was 21.78 (range: 0.92–190.55) months.

### 3.3. Survival in High-Risk Patients Compared with Standard-Risk Patients

**Figure 3:**
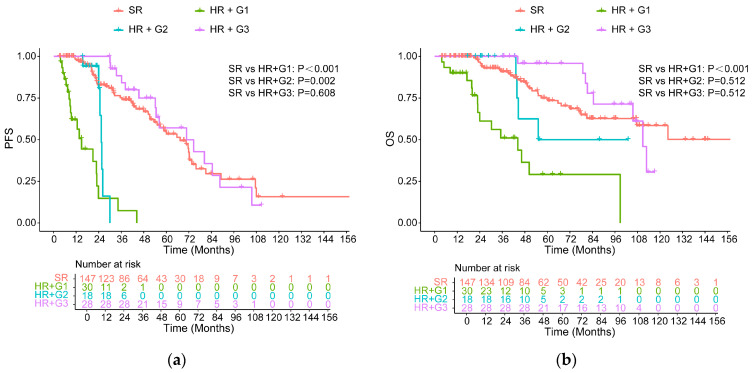
PFS (**a**) and OS (**b**) for all high-risk patients with different durations of MRD negativity versus SR patients.

All high-risk MM patients were classified into three groups on the basis of MRD-negative duration: <1 year, 1–2 years, and ≥2 years, named G1, G2, and G3, respectively. Detailed survival data for every subgroup were provided in Table S1. Among 28 patients with MRD-negative duration of more than 2 years, progression and death occurred in 15 and 8 patients, respectively. The median PFS and OS were 70.77 months (95% CI: 40.78–100.75) and 110.82 months (95% CI: 103.13–118.51), respectively. The survival analysis suggested that high-risk patients who achieved initial MRD negativity for less than 2 years still had significantly inferior PFS compared with the standard-risk group. However, this difference disappeared after high-risk patients maintained MRD negativity for ≥2 years (70.77 vs. 67.38 months, *p* = 0.608, Figure 3a). PFS was significantly longer in patients with an MRD negativity duration of ≥ 2 years compared with those with durations of <1 year (*p* < 0.001) and 1–2 years (*p* = 0.002). OS was also longer in the group with MRD-negative status lasting for over 24 months compared with those with durations of <1 year (*p* < 0.001) and 1–2 years (*p* = 0.049), and there was no significant difference compared with standard-risk patients (110.82 vs. NR, *p* = 0.512). Furthermore, MRD negativity lasting for 1–2 years was also beneficial for OS and had no difference with the standard-risk group (*p* = 0.512, Figure 3b).

**Figure 4:**
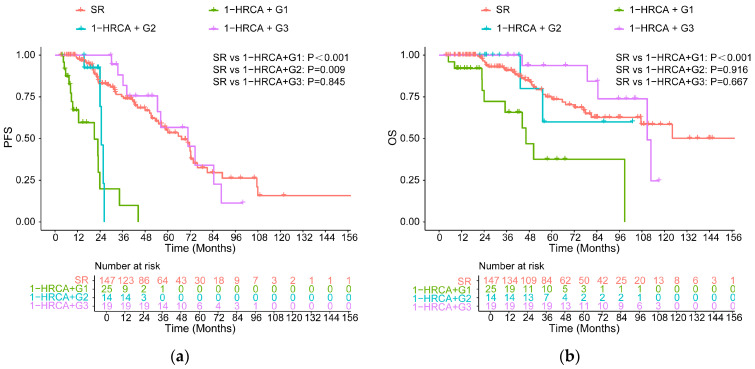
PFS (**a**) and OS (**b**) for high-risk patients carrying a single high-risk cytogenetic abnormality (1-HRCA) with different durations of MRD negativity versus SR patients.

Among 58 patients with 1-HRCA, PFS was also prolonged with persistent MRD-negative status for over 2 years (median PFS, 70.77 months), compared with less than 1 year (vs. 20.90 months, *p* < 0.001) and a duration of 1–2 years (vs. 24.64 months, *p* < 0.001), and was not significantly different from that of the standard-risk group (vs. 67.38 months, *p* = 0.844, Figure 4a). In terms of OS, no statistically significant difference was observed between patients with a duration of more than 2 years and the standard-risk group (110.82 months vs. NR, *p* = 0.667, Figure 4b).

**Figure 5:**
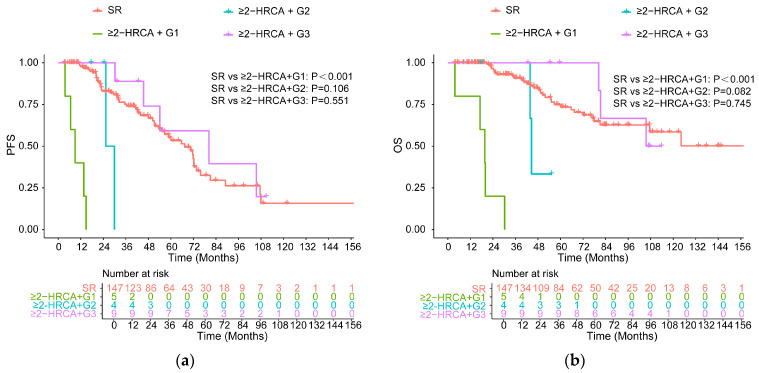
PFS (**a**) and OS (**b**) for high-risk patients carrying two or more high-risk cytogenetic abnormalities (≥2 HRCAs) with different duration of MRD negativity versus SR patients.

For patients with ≥2 HRCAs, statistical comparisons were limited due to the small sample size. The comparison showed that there was no statistically significant difference between patients with a duration of more than 2 years and the standard-risk group for PFS or OS (PFS: *p* = 0.551, OS: *p* = 0.745, Figure 5a,b).

### 3.4. Survival in Patients with Different MRD-Negative Durations

**Figure 6:**
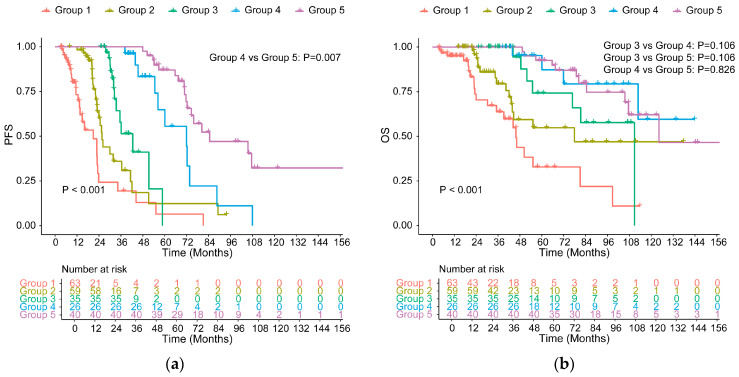
PFS (**a**) and OS (**b**) in all patients with different MRD-negative duration. Groups were as follows (the same below): MRD-negative duration less than 1 year (Group 1), 1–2 years (Group 2), 2–3 years (Group 3), 3–4 years (Group 4) and ≥4 years (Group 5).

All cases were classified into five groups by the duration of MRD negativity: less than 1 year, 1–2 years, 2–3 years, 3–4 years, and ≥4 years, respectively, named Groups 1–5, as shown in Table S2. Detailed patient numbers, events, and median survival times for each group were provided in Table S2. PFS was significantly associated with longer durations of MRD negativity (*p* < 0.001, Figure 6a). No statistically significant differences were observed among Group 3, Group 4, and Group 5 for OS (*p* > 0.100, Figure 6b).

**Figure 7:**
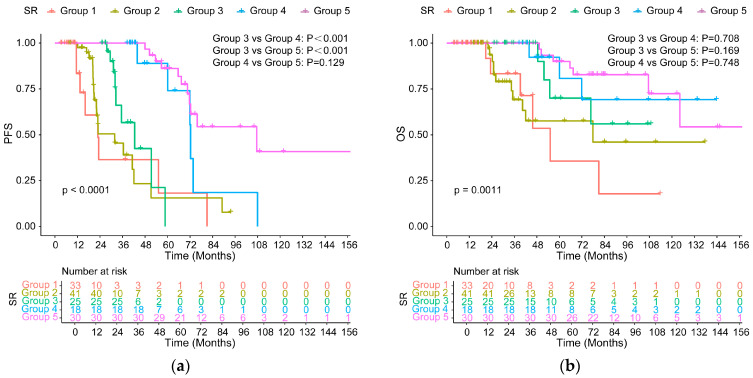
PFS (**a**) and OS (**b**) for SR patients with different MRD-negative durations.

In standard-risk patients, the difference was not significant between Group 4 and Group 5 for PFS (72.25 vs. 107.50 months, *p* = 0.129) and PFS in Group 4 and 5 differed compared with that in the other groups (*p* < 0.01, Figure 7a). OS for patients in Group 5 was similar to Group 3 (*p* = 0.169) and Group 4 (*p* = 0.748, Figure 7b). These results demonstrated that MRD negativity maintained for over 3 years was related to longer PFS and OS in the SR group.

**Figure 8:**
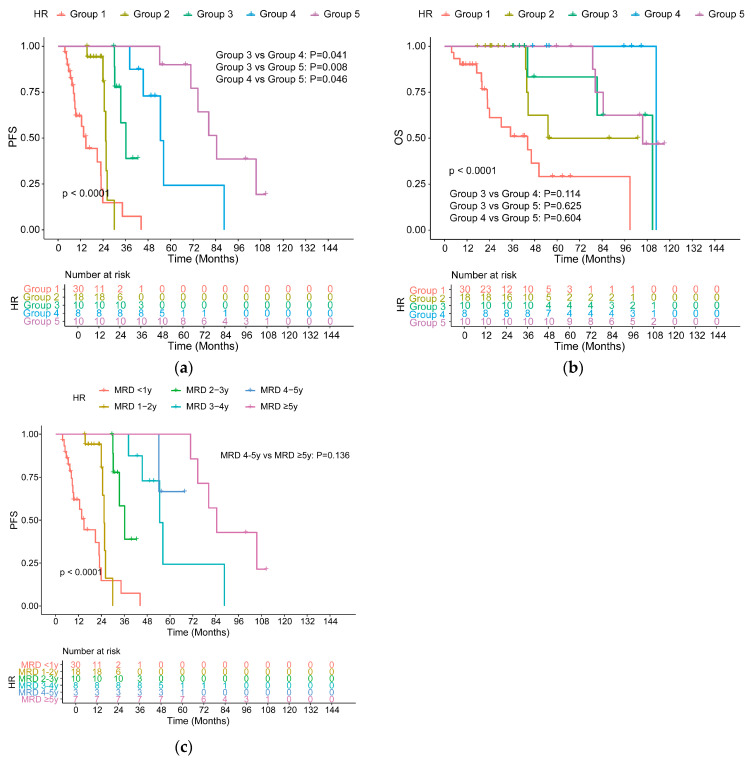
PFS (**a**) and OS (**b**) for HR patients with different MRD-negative durations. PFS (**c**) in HR patients with an MRD-negative duration of 4–5 years and ≥5 years.

High-risk patients in Group 5 had a median PFS of 84.53 months, which was better than that in other groups (*p* < 0.05, Figure 8a). Unlike standard-risk patients, a statistical difference was observed between Group 4 and Group 5 (54.57 vs. 84.53 months, *p* = 0.046). In terms of OS, consistent with the findings in SR patients, no differences were observed among groups with an MRD-negative duration of 2 years or longer (*p* > 0.100, Figure 8b). As an exploratory analysis of the prognostic value of MRD negativity duration for PFS, 10 high-risk patients in Group 5 were divided into patients with a duration lasting for 4–5 years and those with a duration of ≥5 years. The detailed data were provided in Table S2. No statistical difference was found in PFS between the two groups (NR vs. 84.53 months, *p* = 0.136, Figure 8c). It is important to emphasize that the proposed 4-year threshold for high-risk patients is based on only 10 patients. The small number of patients in these longest-duration strata substantially limits the reliability of this threshold.

However, similar results were not observed in 18 patients with ≥2 HRCAs due to the small number of patients in each group. There were no statistically significant differences in PFS among Group 3, Group 4, and Group 5 (*p* > 0.05, Figure S2a). There were no statistically significant differences in OS among Group 2, Group 3, Group 4, and Group 5 (*p* > 0.10, Figure S2b). This 4-year threshold was limited by insufficient statistical power.

### 3.5. Univariate and Multivariate Analyses

In univariate analysis, adverse cytogenetics, amyloidosis (AL), and MRD-negative status for ≥2 years were predictors of PFS. In multivariate analysis, HRCAs (HR = 2.30, 95%CI: 1.50–3.51, *p* < 0.001) were associated with inferior PFS, but an MRD negativity interval of ≥2 years (HR = 0.14, 95%CI: 0.09–0.22, *p* < 0.001) was a beneficial factor for PFS (Table 3). In terms of OS, AL and MRD negativity for ≥2 years both had significant effects, while high-risk cytogenetics showed a relevant trend in univariate analysis. In multivariate analysis of OS, an MRD-negative interval of ≥2 years was the only significant factor (HR = 0.22, 95% CI: 0.13–0.39, *p* < 0.001, Table 3).

## 4. Discussion

Achieving MRD-negative status has been proven to be one of the strongest predictors of favorable prognosis, and MRD negativity is associated with deep remission and a low risk of mortality [24,25]. The prognostic value of consistent MRD detection is more important than that of a single assessment [26]. MRD-driven treatment escalation or de-escalation strategies are being explored in numerous clinical trials, such as MIDAS, PERSEUS, DRAMMATIC, and RADAR [17,27,28]. However, the minimum duration of MRD negativity that can improve the prognosis of MM patients is not well defined, especially in patients with HRCAs [15].

The significance of MRD status for survival in HR patients defined by cytogenetics has no consensus. The retrospective study in our institute reveals that high-risk cytogenetic abnormalities are still correlated with inferior PFS and OS, although these patients received induction, ASCT, and maintenance treatment, which is consistent with previous findings. The study of the Myeloma XI trial indicated that PFS in patients carrying one or more HRCAs was inferior to that of the standard-risk group despite achieving MRD negativity after ASCT [29]. In addition, Li et al. reported a similar result, showing that PFS remained inferior in HR and MRD-negative patients [21].

Nevertheless, our study suggests that long-term maintenance of MRD negativity may improve the adverse prognosis associated with adverse genetic changes. The survival analysis showed that, although high-risk patients continue to show inferior PFS compared with standard-risk patients upon initially achieving MRD negativity, a duration of MRD negativity of ≥2 years was associated with superior PFS and OS, which were not significantly different from those of SR patients. This means durable MRD negativity may alter the baseline risk factors. Several studies showed that persistent MRD negativity lasting for at least 2 years was strongly beneficial for survival [30,31]. Furthermore, this result is consistent with the analysis of the FORTE trial, which suggested that the 4-year PFS was comparable between patients carrying at least 2 HRCAs and having sustained MRD negativity for 1 year and those with 0 or 1 HRCA [32]. Overall, continuous and deep remission may effectively extend the survival of high-risk patients and provide guidance for individual therapy in high-risk MM patients with persistent MRD negativity.

Moreover, an exploratory finding in our study is that maintaining MRD negativity for more than 4 years appeared to be associated with prolonged PFS in the high-risk group, while this interval was 3 years in standard-risk patients. This is similar to our finding that high-risk cytogenetics were related to poor prognosis for patients with consistent MRD negativity. It is also important to emphasize that the high-risk subgroup with MRD negativity for ≥4 years comprised only 10 patients, and the 4-year threshold should be regarded as a preliminary, exploratory marker rather than a validated clinical decision point. In addition, maintaining 2-year MRD negativity was linked to longer OS regardless of risk group. However, in the exploratory subgroup of patients with ≥2 HRCAs, the analysis suggested that MRD negativity duration was 2 years for PFS and only 1 year for OS. Due to the limited cases of patients with ≥2 HRCAs, the reliability of this finding is insufficient and needs further confirmation in ultra-high-risk patients. Previous studies have reported that a longer duration of MRD negativity is correlated with better clinical outcomes. San Miguel et al. conducted a summary analysis of the MAIA and ALCYONE trials, which demonstrated that MRD-negative durations of ≥6 months or 12 months were associated with favorable PFS [12]. Wang et al. indicated a duration of ≥2 years was associated with superior PFS compared with a duration of ≥1 year, despite no statistical difference [33]. MRD negativity over 24 months suggested longer PFS and OS [34]. In another study, MRD negativity lasting for more than 3 years had important prognostic value [35]. All of the above suggest that durable MRD negativity is a critical factor associated with good outcomes. This study provides a theoretical reference for minimum duration of MRD negativity related to superior prognosis.

However, there are some limitations to the study. Firstly, because our research was a retrospective analysis at a single center, the treatment regimens and processes of patients were not entirely identical, and a limited number of cases were enrolled. These results should not be used to guide clinical decision-making. Secondly, the follow-up frequency and MRD detection time points were not set uniformly in advance due to the retrospective data collection. Furthermore, the sensitivity of MRD detection was relatively low because of restrictions in the detection method, especially because some data from early years lacked sophisticated detection techniques, so bias was inevitable in this study. In addition, our finding was based on MRD detection sensitivity of 10^−5^, and deeper sensitivity may be associated with similar or even superior outcomes, which requires future studies using more sensitive detection methods for validation, such as NGF and NGS. Non-transplant patients and patients with imaging-positive MRD-negative status were not included. Additionally, the ‘duration of MRD negativity’, as a complex time-dependent variable, was related to possible bias. Although we made a plan for addressing this bias, the inherent limitation of a single-center retrospective design cannot be fully eliminated. Our study provides theoretical support for subsequent research. Furthermore, several subgroup analyses that lacked statistical power and strong reliability should be considered exploratory analyses, such as the 4–5 years versus ≥5 years MRD-negative duration in high-risk patients and all analyses for patients with ≥2 HRCAs. It is important to identify the existence of selection bias in our cohort. Only 36.8% of high-risk patients maintained MRD negativity for ≥2 years, and this proportion was less than that in the standard-risk group. This indicates that durable MRD negativity is harder to achieve in high-risk patients and that high-risk patients with poor prognosis may not have been included in the subgroup with ≥2-year MRD negativity. This may overestimate the effect of sustained MRD negativity.

Therefore, larger numbers of patients with more than one HRCA are needed to confirm whether an even longer MRD duration threshold is required. Moreover, future prospective clinical trials are necessary and should include more available MRD data from multiple centers. This is helpful for exploring the appropriate MRD-negative duration and determining whether MRD duration can serve as a reliable biomarker for developing individualized strategies.

## 5. Conclusions

In conclusion, our study identified that durable MRD-negative status improved survival in patients with HRCAs; specifically, a duration of more than 2 years was associated with outcomes approaching those of standard-risk patients in this cohort, and an interval of more than 4 years may be correlated with additional PFS benefit in high-risk patients. This study emphasized the value of MRD dynamic evaluation and persistent MRD negativity in the prognostic monitoring of MM patients. However, this finding was based on a limited number of patients and requires confirmation in larger and prospective cohorts before it can be used to guide treatment decision.

## Figures and Tables

**Table 1:** Baseline characteristics of the 223 NDMM patients.

Characteristic	Number or Median	Ratio (%)/Range
Age (years)	56	26–69
Sex (n)		
Male	125	56.05
Female	98	43.95
Monoclonal protein type (n)		
IgG	116	52.02
IgA	53	23.77
IgM	1	0.45
IgD	7	3.14
Light chain disease	46	20.63
R-ISS stage (n)		
I	52	23.74
II	131	59.82
III	36	16.44
Ca (mmol/L)	2.36	1.60–3.64
Hb (g/L)	96	44–159
Cr (umol/L)	84.2	16.0–1213.0
Bone marrow plasma cells (%)	23.00	1.00–91.50
β2-MG (ug/L)	3351.11	1008.0–24,582.2
LDH (U/L)	169	75–4759
ALB (g/L)	35.4	14.7–49.0
Cytogenetic abnormality (n)		
SRCA	147	65.92
1-HRCA	58	26.01
≥2-HRCA	18	8.07
del(17p)	11	4.93
t(4;14)	38	17.04
t(14;16)	5	2.24
del(1p32)	9/68	13.24
1q21amp	35/212	16.51
Amyloidosis (n)	25/222	11.26
HBsAg (n)	35/222	15.77
EMD (n)	56/222	25.23
Tandem ASCT (n)	16	7.17
Induction regimen		
PIs based	189	84.75
Others	34	15.25
Maintenance regimen		
IMiDs based	138/220	62.73
PIs based	27/220	12.27
Dara based	10/220	4.55
Others	45/220	20.45

**Table 2:** Comparison of basic clinical information from MRD negativity groups.

Variables	MRD-Negative Duration		*p* Value
	<2 Years (n = 122)	≥2 Years (n = 101)	
Age, n (%)			0.495
<65 years	105 (86.07)	90 (89.11)	
≥65 years	17 (13.93)	11 (10.89)	
Sex, n (%)			0.917
Male	68 (55.74)	57 (56.44)	
Female	54 (44.26)	44 (43.56)	
Monoclonal protein type, n (%)			0.917
IgG	63 (51.64)	53 (52.48)	
IgA	31 (25.41)	22 (21.78)	
IgM	1 (0.82)	0	
IgD	4 (3.28)	3 (2.97)	
Light chain	23 (18.85)	23 (22.77)	
R-ISS stage, n (%)			0.912
I	28 (23.14)	24 (24.49)	
II	72 (59.50)	59 (60.20)	
III	21 (17.36)	15 (15.31)	
Bone marrow plasma cells (%, median)	22.5	24	0.721
Ca, n (%)			0.123
≤2.75 mmol/L	101 (84.17)	92 (91.09)	
>2.75 mmol/L	19 (15.57)	9 (8.91)	
Hb, n (%)			0.379
<100 g/L	70 (57.38)	52 (51.49)	
≥100 g/L	52 (42.62)	49 (48.51)	
Cr, n (%)			0.123
<177 umol/L	103 (84.43)	83 (82.18)	
≥177 umol/L	19 (15.57)	18 (17.82)	
ALB, n (%)			0.749
<35 g/L	58 (48.33)	51 (50.50)	
≥35 g/L	62 (51.67)	50 (49.50)	
LDH, n (%)			0.178
≤240 U/L	95 (79.83)	85 (86.73)	
>240 U/L	24 (20.17)	13 (13.27)	
β2-MG, n (%)			0.176
<3500 ug/L	60 (50.85)	60 (60.00)	
≥3500 ug/L	58 (49.15)	40 (40.00)	
Cytogenetic abnormality, n (%)			0.083
SRCA	74 (60.66)	73 (72.28)	
1-HRCA	39 (31.97)	19 (18.81)	
≥2-HRCA	9 (7.37)	9 (8.91)	
AL, n (%)	17 (14.05)	8 (7.92)	0.15
EMD, n (%)	30 (24.79)	26 (25.74)	0.871
HBsAg, n (%)	20 (16.53)	15 (14.85)	0.733
Tandem ASCT, n (%)	11 (9.02)	5 (4.95)	0.242
Induction regimen			0.006
PIs based	96 (78.69)	93 (92.08)	
Others	26 (21.31)	8 (7.92)	
Maintenance regimen			0.277
PIs based	14 (11.48)	13 (12.87)	
IMiDs based	70 (57.38)	68 (67.33)	
Dara based	6 (4.92)	4 (3.96)	
Others	32 (26.23)	16 (15.84)	

**Table 3:** Univariate and multivariate analyses of PFS and OS for enrolled patients.

PFS	Univariate Analysis			Multivariate Analysis		
	HR	95% CI	*p* Value	HR	95% CI	*p* Value
Age (≥65 years)	1.05	0.52–2.09	0.899			
Sex (female)	0.70	0.46–1.07	0.099			
Plasma cell	1.01	1.00–1.02	0.072			
R-ISS stage (III)	1.33	0.80–2.21	0.268			
Cytogenetics (High-risk)	1.94	1.29–2.91	0.002	2.26	147–3.48	<0.01
Ca (>2.75 mmol/L)	1.46	0.82–2.59	0.200			
Creatinine (≥177 umol/L)	1.04	0.62–1.74	0.890			
Hb (≥100 g/L)	0.85	0.57–1.29	0.452			
ALB (≥35 g/L)	0.88	0.59–1.33	0.553			
β2-MG (≥3.5 mg/L)	1.20	0.79–1.80	0.392			
LDH (>240 U/L)	1.36	0.80–2.32	0.251			
Amyloidosis	2.00	1.10–3.62	0.022	1.37	0.75–2.49	0.309
Extramedullary disease	1.10	0.69–1.73	0.696			
Tandem ASCT	1.30	0.47–3.57	0.613			
Induction regimen (others)	1.55	0.82–2.95	0.178			
Maintenance regimen						
PIs based						
IMiDs based	0.91	0.45–1.85	0.799			
Dara based	1.72	0.53–5.65	0.369			
Others	1.00	0.45–2.20	0992			
MRD-negativity ≥ 2years	0.16	0.10–0.25	<0.01	0.14	0.09–0.22	<0.01
**OS**	**Univariate Analysis**			**Multivariate Analysis**		
	**HR**	**95% CI**	***p*** **Value**	**HR**	**95% CI**	***p*** **Value**
Age (≥65 years)	1.48	0.67–3.27	0.337			
Sex (female)	0.93	0.55–1.57	0.782			
Plasma cell	1.01	1.00–1.02	0.108			
R-ISS stage (III)	1.76	0.95–3.27	0.075			
Cytogenetics (High-risk)	1.77	1.05–2.97	0.033	1.57	0.93–2.66	0.094
Ca (>2.75 mmol/L)	1.71	0.84–3.48	0.141			
Creatinine (≥177 umol/L)	1.24	0.64–2.40	0.517			
Hb (≥100 g/L)	1.06	0.63–1.77	0.831			
ALB (≥35 g/L)	0.80	0.48–1.35	0.409			
β2-MG (≥3.5 mg/L)	1.52	0.91–2.55	0.111			
LDH (>240 U/L)	1.49	0.78–2.82	0.224			
Amyloidosis	2.80	1.38–5.64	0.004	1.99	0.99–4.03	0.055
Extramedullary disease	1.38	0.78–2.43	0.272			
Tandem ASCT	0.59	0.08–4.31	0.603			
Induction regimen (others)	1.24	0.53–2.93	0.620			
Maintenance regimen						
PIs based						
IMiDs based	1.58	0.56–4.46	0.383			
Dara based	0		0.996			
Others	1.94	0.64–5.86	0.243			
MRD-negativity ≥ 2 years	0.22	0.13–0.39	<0.01	0.25	0.14–0.43	<0.01

## Data Availability

The datasets generated during the current study are available from the corresponding author on reasonable request.

## Supplementary Materials

The following supporting information can be downloaded at: https://www.mdpi.com/article/10.3390/cancers18101569/s1, Figure S1: The MRD monitoring timepoints. Figure S2: PFS (a) and OS (b) for ≥2 HRCAs patients with different MRD-negative duration. Table S1: Subgroup analysis of high-risk patients according to number of HRCA and MRD negativity duration: patient numbers, events, and median survival. Table S2: All cases were classified into five groups by the duration of MRD negativity: less than 1 year, 1–2 years, 2–3 years, 3–4 years and ≥4 years, respectively, named as Group 1–5. Subgroup analyses according to MRD negativity duration: patient numbers, events, and median survival.

## Abbreviations

The following abbreviations are used in this manuscript:

MM	Multiple myeloma
NDMM	Newly diagnosed multiple myeloma
ASCT	Autologous hematopoietic stem cell transplantation
R-ISS	Revised International Staging System
HRCA	High-risk cytogenetic abnormality
MRD	Minimal residual disease
PFS	Progression-free survival
OS	Overall survival
SR	Standard-risk
HR	High-risk
