# Supplementary material for: Sustained MRD Negative for 4 Years Is a Significant Marker of Prognosis in Patients with High-Risk Multiple Myeloma
Source: Cancers (Basel). 2026 May 12;18(10):1569. doi: 10.3390/cancers18101569 (PMC13204074; doi:10.3390/cancers18101569)
Supplement: Supplementary file 1 [file cancers-18-01569-s001.zip › cancers-4262566-supplementary.pdf]

cancers

MDPI

Supplementary Materials: Sustained MRD Negative for 4 Years Is a Significant Marker of Prognosis in Patients with High-Risk Multiple Myeloma

Huan Liu Meilan Chen, Xiaozhe Li, Lifen Kuang, Jingxia Li, Yanjuan Li and Juan Li

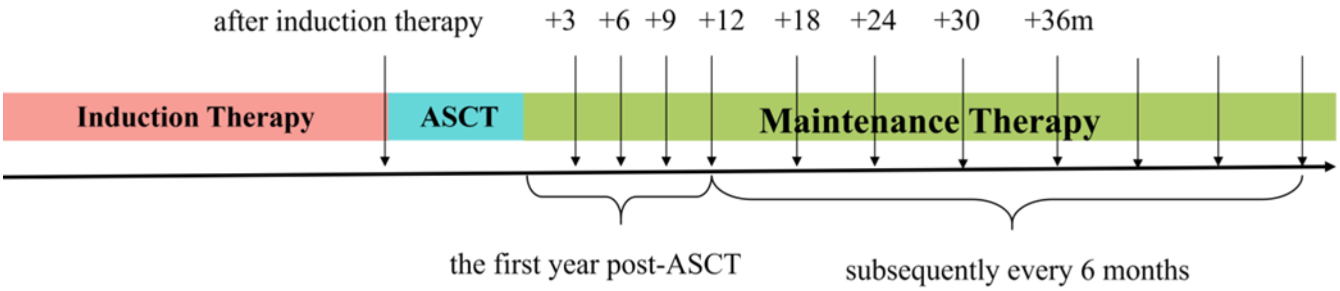

Figure S1. The MRD monitoring timepoints.

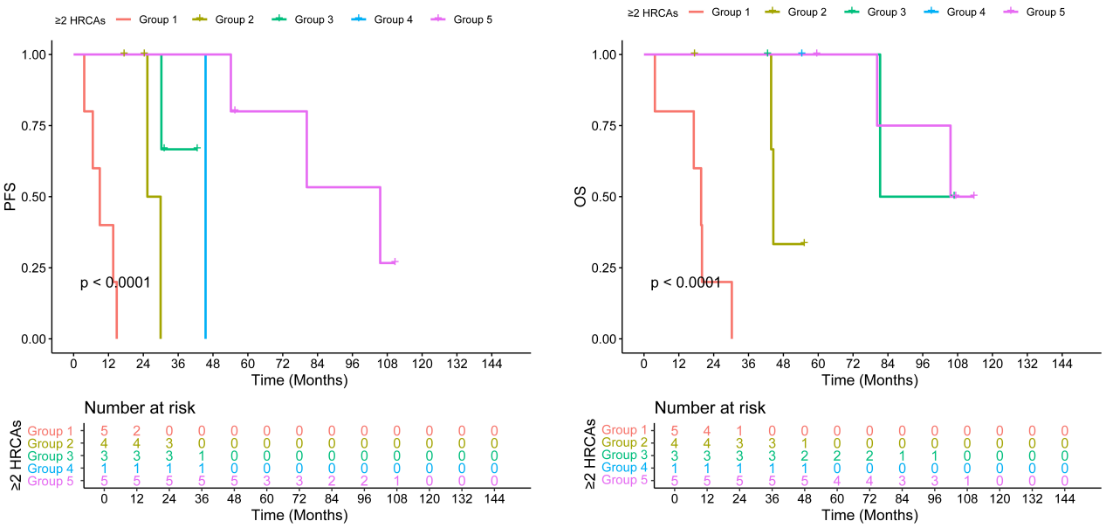

(a) (b)

Figure S2. PFS (a) and OS (b) for ≥2 HRCAs patients with different MRD-negative duration.

Table S1. Subgroup analysis of high-risk patients according to number of HRCA and MRD negativity duration: patient numbers, events, and median survival.

Subgroup	MRDneg duration	No. of patients	PFS events	Median PFS (months, 95% CI)	OS events	Median OS (months, 95% CI)
Standard-risk	-	147	53	67.38(52.68-82.09)	31	NR
High-risk	<1 year (G1)	30	19	14.85(10.69-19.01)	15	44.29(18.67-69.90)
	1–2 years (G2)	18	7	25.40(24.20-26.60)	4	55.13
	≥2 years (G3)	28	15	70.77(40.78-100.75)	8	110.82(103.13-118.51)
1-HRCA	<1 year (G1)	25	14	20.90(7.50-34.29)	10	46.23(37.38-55.07)
	1–2 years (G2)	14	5	24.64(22.95-26.34)	2	NR
	≥2 years (G3)	19	10	70.77(46.11-71.31)	5	110.82(86.06-135.57)
≥2 HRCAs	<1 year (G1)	5	5	9.00(3.85-14.15)	5	19.68(14.25-25.11)
	1–2 years (G2)	4	2	27.68	2	44.50(43.34-45.76)

Subgroup	MRDneg duration	No. of patients	PFS events	Median PFS (months, 95% CI)	OS events	Median OS (months, 95% CI)
	≥2 years (G3)	9	5	80.30(27.63-132.96)	3	109.59

Table S2. All cases were classified into five groups by the duration of MRD negativity: less than 1 year, 1-2 years, 2-3 years, 3-4 years and ≥ 4 years, respectively, named as Group 1-5. Subgroup analyses according to MRD negativity duration: patient numbers, events, and median survival.

Subgroup	MRDneg duration	No. of patients	PFS events	Median PFS (months, 95% CI)	OS events	Median OS (months, 95% CI)
All patients	Group 1	63	27	20.90(10.38-31.41)	21	45.80(37.25-54.35)
	Group 2	59	26	25.66(23.41-27.91)	15	77.90
	Group 3	35	13	42.48(31.58-53.38)	7	110.82
	Group 4	26	11	71.89(40.12-103.65)	4	NR
	Group 5	40	17	84.53(43.91-68.65)	11	124.22
Standard-risk	Group 1	33	8	22.83(13.15-32.52)	6	55.13(38.30-71.96)
	Group 2	41	19	31.80(15.15-48.45)	11	77.90
	Group 3	25	9	42.48(26.66-58.31)	4	NR
	Group 4	18	6	72.25(71.47-73.02)	3	NR
	Group 5	30	11	107.50(58.22-156.77)	7	NR
High-risk	Group 1	30	19	14.85(10.69-19.01)	15	44.29(18.67-69.90)
	Group 2	18	7	25.40(24.20-26.60)	4	79.03
	Group 3	10	4	36.17(30.50-41.85)	3	110.82
	Group 4	8	5	54.47(44.65-64.49)	1	112.79
	Group 5	10	6	84.53(71.35-97.72)	4	105.56
≥2 HRCAs	Group 1	5	5	9.00(3.85-14.15)	5	19.68
	Group 2	4	2	27.68	2	44.50
	Group 3	3	1	NR	1	NR
	Group 4	1	1	45.44	0	NR
	Group 5	5	3	105.56(62.90-148.22)	2	NR
